# Aberrant mesenchymal differentiation of glioma stem-like cells: implications for therapeutic targeting

**DOI:** 10.18632/oncotarget.5219

**Published:** 2015-08-19

**Authors:** Veerakumar Balasubramaniyan, Brian Vaillant, Shuzhen Wang, Joy Gumin, M. Elena Butalid, Ke Sai, Farah Mukheef, Se Hoon Kim, H.W.G.M. Boddeke, Frederick Lang, Kenneth Aldape, Erik P. Sulman, Krishna P. Bhat, Howard Colman

**Affiliations:** ^1^ Department of Neuro-Oncology, University of Texas, MD Anderson Cancer Center, Houston, Texas, USA; ^2^ Department of Translational Molecular Pathology, University of Texas, MD Anderson Cancer Center, Houston, Texas, USA; ^3^ Department of Neurosurgery, University of Texas, MD Anderson Cancer Center, Houston, Texas, USA; ^4^ Brain Tumor Center, University of Texas, MD Anderson Cancer Center, Houston, Texas, USA; ^5^ Department of Neuroscience, University of Groningen, University Medical Center Groningen, Groningen, The Netherlands; ^6^ Department of Pathology, Toronto General Hospital/Princess Margaret Cancer Centre, Toronto, Ontario; ^7^ Department of Radiation Oncology, University of Texas, MD Anderson Cancer Center, Houston, Texas, USA; ^8^ Department of Neurosurgery, Huntsman Cancer Institute, University of Utah, Salt Lake City, Utah, USA; ^9^ Department of Neuroscience, University of Groningen, University Medical Center Groningen, Groningen, The Netherlands

**Keywords:** glioblastoma, glioma stem-like cells, serum differentiation, mesenchymal, tumorigenicity

## Abstract

Differentiation has been proposed as a therapeutic strategy for glioblastoma (GBM) in part due to observations of stem-like cells in GBM that have been shown to undergo terminal differentiation in response to growth factor withdrawal and BMP activation. However, the effects of long term exposure to serum culture conditions on glioma sphere cultures/glioma stem-like cells (GSCs) have not been examined. Here we show that GSCs retained both neurosphere formation and tumor initiation abilities after short or long term serum exposure. Under these conditions, GSCs expressed both neural lineage and stem cell markers, highlighting the aberrant pseudo-differentiation state. GSCs maintained under adherent serum cultured conditions continued to proliferate and initiate tumor formation with efficiencies similar to GSCs maintained under proliferating (neurosphere) conditions. Proneural (PN) GSCs under serum exposure showed an induction of mesenchymal (MES) gene expression signatures. Our data indicate that exposure to serum containing media result in aberrant differentiation (e.g. toward MES lineage) and activation of alternative oncogenic pathways in GSCs.

## INTRODUCTION

Glioblastoma (GBM) is the most common malignant primary central nervous system tumor in adults. Despite scientific and clinical advances, GBM is highly resistant to current therapies and remains essentially incurable [1]. The complex heterogeneity of GBM is evidenced by numerous genomic studies showing distinct molecular entities in GBM, despite a single histological classification [2, 3]. In addition, epigenomic studies have identified multiple epigenetic subtypes in GBM, including the glioma methylator phenotype (G-CIMP) that correlates with better overall survival and IDH mutation status [4].

Recent evidence supports the existence of cancer stem cells in many solid tumors including GBM. The cancer stem cell hypothesis postulates that tumor initiation and recurrence is dependent on a small subset of cells with stem cell like properties including multi-lineage differentiation potential and indefinite self-renewal [5-7]. Consistent with this hypothesis, glioma sphere cultures/glioma stem-like cells (GSCs) have been successfully isolated and expanded from human tumors using serum-free “neurosphere” culture methods pioneered in studies of neural stem cell biology [8-11]. Initial studies identified CD133 as a putative marker for GSCs [11, 12]; however, recent studies indicate that CD133 negative stem cells also harbor tumor initiating capacity [13, 14] and additional markers such as SSEA-1/CD-15, L1CAM, A2B5, CD90 and CD44 have been proposed [13, 15-17]. While widely used as a method for expanding stem cells of neural origin, neurosphere cultures can consist of a heterogeneous mixture of cells with stem cell-like properties as well as clonally derived progeny and differentiated cells [18, 19]. The observed molecular heterogeneity of human GBMs has, at least in part, been attributed to the multi-lineage differentiation property of GSCs.

We recently showed that GSCs can differ in their gene expression and molecular profiles, and that these molecular differences are associated with differing biological properties and treatment responsiveness [20]. Prior studies have identified 3-5 major gene expression subtypes of GBM, with the most consistent phenotypes usually referred to as proneural (PN) and mesenchymal (MES) based on their resemblance to normal neural or extracellular matrix tissues [2,3]. Similar observations have held true in studies involving GSCs, with a consensus that at least two subtypes (PN and MES) exist [21, 22]. Furthermore, we have found that a subset of the PN GSCs undergo differentiation to a MES state in a TNF-α/NF-κB-dependent manner with an associated enrichment of CD44 expressing subpopulations and this can be regulated by the tumor microenvironment [20]. Thus it appears that the transcriptome signatures and biological properties of GSCs could be influenced by the tumor microenvironment. Alternatively, a PN to MES shift was shown in murine tumors upon exposure to radiation without influence of the stromal compartment [23]. These observations coupled with recent reports showing intratumoral heterogeneity of gene expression patterns [24] suggests that cell intrinsic and extrinsic factors can influence cancer stem cell properties and differentiation state.

Prior studies have demonstrated that upon withdrawal of growth factors or addition of serum, GSCs express markers associated with neural lineages (neurons or glia), similar to multipotent normal neural stem cells (NSCs). This *in vitro* differentiation capacity is often used as one of the criteria to define cancer stem cells [10, 25]. Furthermore, differentiation has been implicated as a potential therapeutic approach to reduce GSC tumorigenicity [26-29]. Acutely dissociated cells cultured directly from human GBM tumors as adherent cultures in serum containing media were not tumorigenic, while cells from the same tumor expanded as neurospheres demonstrated characteristics of GSCs [10]. Several studies have also identified culture conditions or treatments of GSCs targeting specific pathways involved in neural development and differentiation that can reduce tumor initiation in mice [26, 27, 30]. While these data suggest that key developmental pathways may be important regulators of GSC self-renewal and tumorigenicity, the long term effects of serum exposure on GSCs have not been examined. The present study was aimed at examining the *in vitro* and *in vivo* alterations on culturing GSCs in serum containing media.

## RESULTS

### Characterization of GSCs derived from human GBM

**Figure 1:**
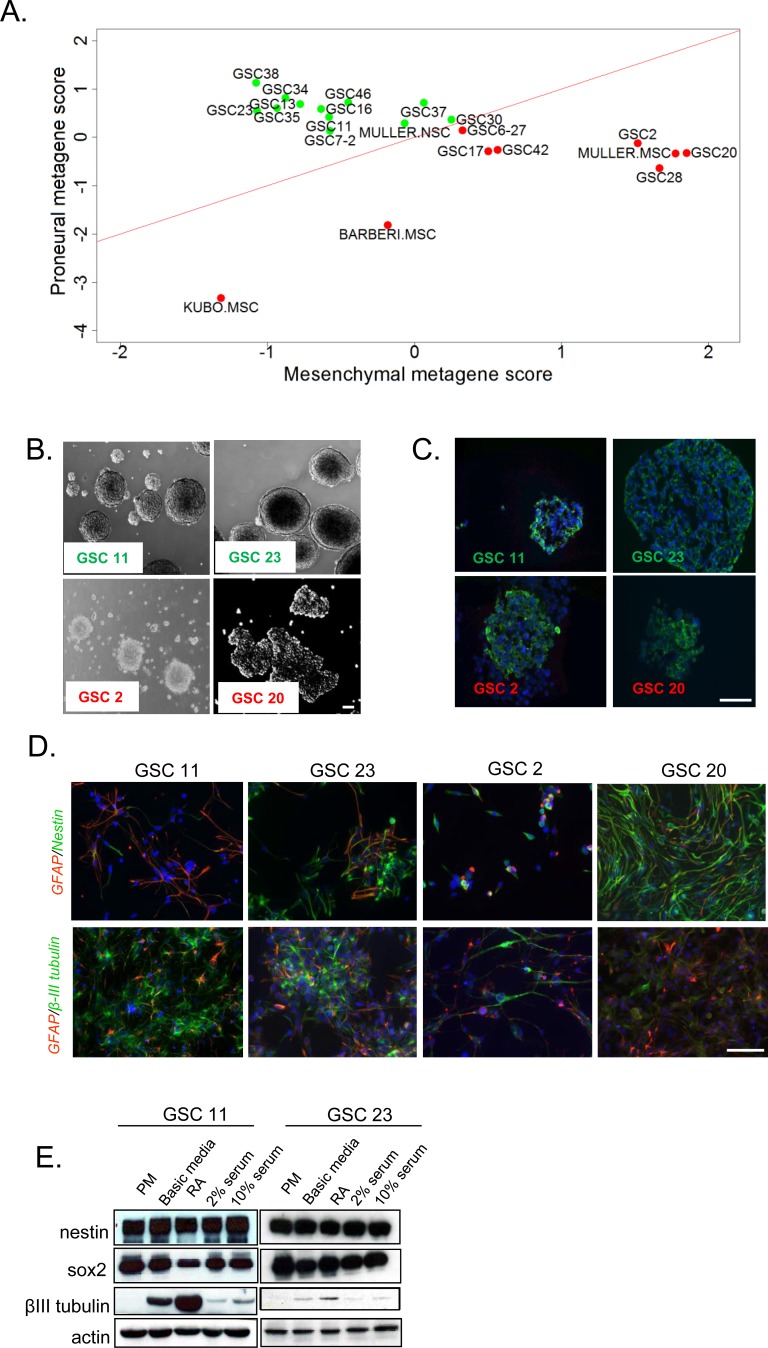
*In vitro* evaluation of stem cell like-characteristics of human GSCs **A.** Metagene plots of PN (green) versus MES (red) GSCs in comparison to human normal NSCs and MSCs. **B.** Bright field microscopic images of neurospheres of GSCs 11, 23, 2, and 20. Scale bar = 50μm. **C.** Undifferentiated GSC neurospheres stained for NSC marker nestin. Scale bar = 50μm. **D.** Immunofluorescent analyses of GSCs cultured in media containing retinoic acid or serum. Upper panel shows co-staining for astrocyte marker GFAP (red) and NSC marker nestin (green). Lower panel shows staining for neuronal specific β-III tubulin (green) and astrocytic marker GFAP (red). Scale bar = 100μm. **E.** Western blot analysis of neural stem cell and lineage markers in PN GSCs cultured in PM, basic media that lacks growth factors, RA, 2% serumor 10% serum. β-actin was used as a loading control.

To extend our recent molecular characterization on GSCs, we compared the global gene expression signatures of these cell types against published human NSC and MES stem cell (MSC) datasets. The classification of these GSCs based on a metagene (see methods for details) successfully clustered PN GSCs (shown in green) alongside human NSCs, whereas MES GSCs (red) clustered with the human MSCs (Figure 1A) [20].

It is well established that normal NSCs grow as neurospheres and differentiate into astrocytic, neuronal and oligodendrocytic lineages upon growth factor withdrawal and cytokine exposure [31]. To extend our comparisons beyond gene expression signatures, we closely examined the morphology of the GSCs. Consistent with a NSC-like signature, the PN GSCs (GSC11 and 23) grew as compact neurospheres whereas the MES GSCs (GSC2 and 20) grew as loose clusters (Figure 1B). In spite of these morphological differences, immunocytochemical analysis of neural stem cell marker nestin showed no variable expression amongst GSCs (Figure 1C).

To investigate their differentiation capacities, GSCs were subjected to various culture conditions known to promote differentiation. GSCs were seeded onto poly-d-lysine/laminin coated coverslips or plates, and cultured in either basic media (devoid of growth factors), in the presence of all-trans retinoic acid (RA) or varying percentages of serum. After 7-10 days of *in vitro* culture under these conditions, cells were fixed and analyzed for neural lineage markers including GFAP (astrocytes), β-III tubulin (neurons) or nestin (undifferentiated cells) using immunofluorescence. All four GSC lines expressed lineage specific markers (Figure 1D) to varying degrees. In contrast to what is normally observed in NSCs [32], some GSCs co-expressed both GFAP and nestin. The MES GSC20, and to a lesser extent GSC2, retained expression of nestin but were resistant to neural lineage differentiation even after exposure to retinoic acid (RA, Figure 1D, bottom panel), indicating that MES GSCs are more resistant to differentiate towards neural lineages than PN GSCs. Next we examined the expression pattern of these markers by western blotting [3, 5-7]. Short-term (13 days) RA or serum exposure resulted in no alteration of stem/progenitor cell marker nestin, but a variable reduction of SOX2 in western blot assays (Figure 1E). Total levels of the neuronal marker β-III tubulin, was strongly induced in all differentiation paradigms in PN GSCs (Figure 1E).

### Short term RA or serum exposed GSCs retain tumor initiating ability

**Figure 2:**
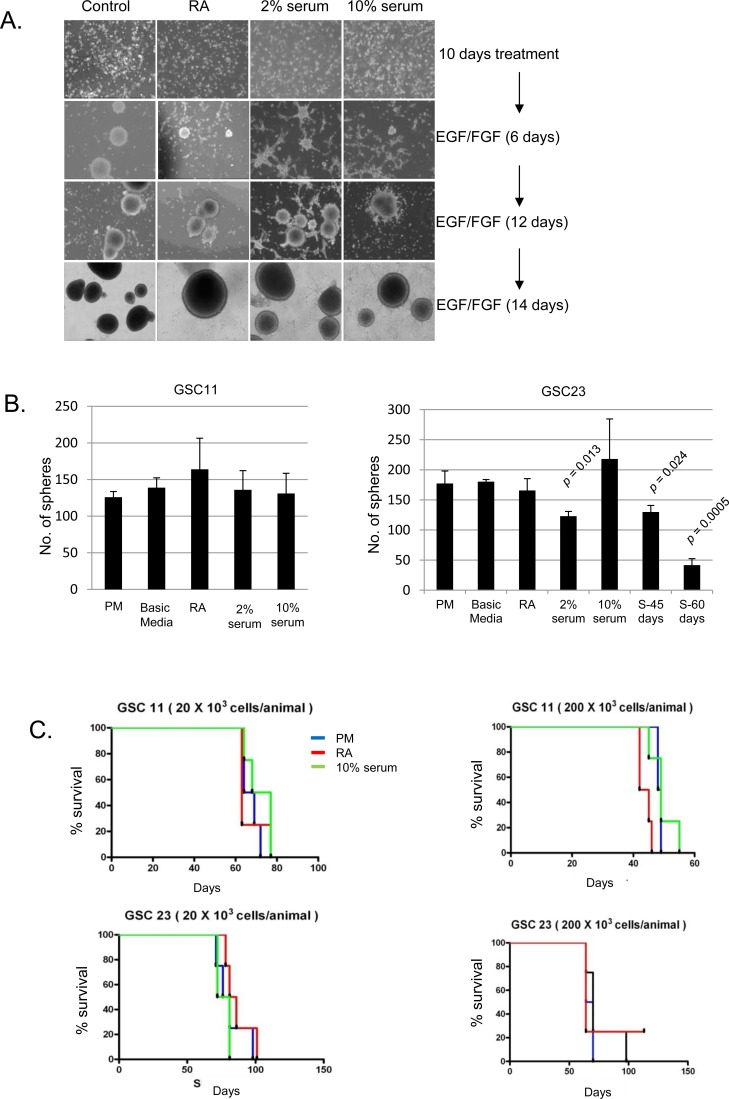
**A.** Brightfield images of GSC11 showing reformation of neurospheres when culturedin PM after 10 days of differentiation in RA, 2% serum or 10% serum. **B.** Neurosphere formation efficiency after 13 days differentiation of GSC11and 23 under various differentiation conditions. Long term differentiation in serum was designated as S-45 (cultured for 45-days of10% serum) or S-60 (60 days in 10% serum. After various differentiationparadigms, cells were plated in triplicate into 96-well plates at 10 cells/well. Bar graphs indicate average of three independent experimentsand error bars represent standard deviation of the mean. A two tailed t-test was performed to test for statistical significance. Comparison was made for all differentiation conditions against neurosphere number in PM **C.** Kaplan Meier curves show the survival of mice implanted with GSC11 or 23 after culturing in PM, RA or 10% serum. *n* = 4 mice per group.

We next sought to examine the impact of short term RA or serum exposure on the self-renewal and tumorigenicity of GSCs. While aberrant differentiation has also been observed in the cancer stem cells isolated from pediatric brain tumors and mouse models of GBM [33, 34], differentiation in normal NSCs typically results in exit from the cell cycle. We thus examined the relationship between expression of lineage markers and cell proliferation as determined by synthesis of DNA during the S phase of the cell cycle by Brdu incorporation in GSCs. To our surprise, a number of proliferating cells co-labeled with lineage markers like GFAP after differentiation induction (Supplementary Figure 1A). To test whether GSCs retained the ability to re-form neurospheres after exposure to differentiating conditions, we switched these cells (after 13 days) to basic media with growth factors (proliferation media, PM). Within three days, GSCs started to aggregate and form free floating neurospheres (Figure 2A). With the exception of GSC23 grown in 2% FBS (which showed a modest decrease), we observed no significant differences in neurosphere formation efficiency between GSCs cultured in differentiating or proliferating conditions (Figure 2B), indicating that short term exposure to RA or serum does not completely abolish self-renewal of GSCs.

Tumor initiation in xenograft models is a defining feature of cancer stem cells. Since our data suggested that serum cultured GSCs continue to divide and maintain neurosphere initiation capacity, we examined if the tumorigenic potential of these GSCs differed from isogenic GSC cultures maintained in PM. To test tumorigenicity, we performed intracranial injections of RA treated and serum cultured populations using high and low cell numbers (2×10^5^ and 2×10^4^ cells per animal respectively). Consistent with our *in vitro* observations, GSCs cultured in all conditions were capable of tumor initiation at comparable rates (Figure 2C). With the exception of RA treated GSC11 (implanted at 2×10^5^ cells/mice), log-rank analysis demonstrated no significant change in the survival times of animals implanted with GSCs cultured under differing conditions (Supplementary Figure 1B). Pathologic examination of these tumors revealed that the tumors from 10% serum (10F) and RA treated GSCs retained the hallmarks of high grade glioma (pseudo-palisading necrosis, Supplementary Figure 1C). Taken together, these results strongly suggest that while exposure of GSCs to RA or serum results in increased expression of lineage restricted markers, these cells also retain key properties of tumor stem cells including neurosphere formation and tumor initiation capacity after short-term culture under these conditions. Moreover, the tumors were indistinguishable when tested for the expression of various markers including nestin, GFAP and OLIG2 by immunohistochemistry (IHC, Supplementary Figure 1D).

### Long term serum exposure induces a MES shift in GSCs

**Figure 3:**
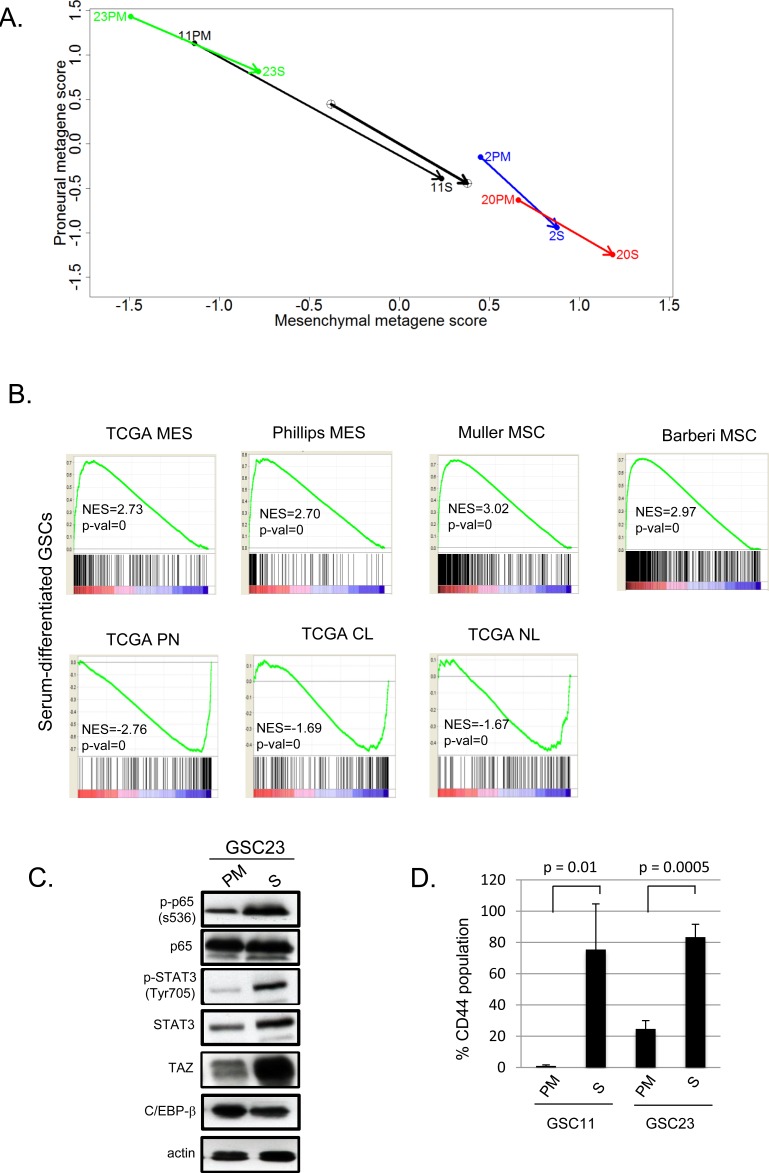
**A.** Metagene plots of GSCs cultured in PM or 10% FBS for 60 days (S). **B.** GSEA analysis of genes induced after differentiation in 10% FBS for 60 days versus queried gene lists. The normalized enrichment scores (NES) and *p* values are shown below each plot. **C.** Western blotting of GSC23 cultured in PM or S. **D.** Bar graphs showing percent CD44 expression in GSCs cultured in PM or S as determined by flow cytometry. Error bars indicate standard deviation of three independent experiments. A two tailed t-test was performed to test for statistical significance.

In order to examine the long term effects of these paradigms, we cultured GSC11 and GSC23 in serum containing media for >60 days. As shown in Figure 2B, neurosphere formation efficiency of adherent cultures in serum were significantly reduced upon reverting to PM when compared to those continuously maintained in PM. Next, to better understand molecular alterations associated with serum culture, we examined global transcriptomic changes of GSCs cultured in these conditions. Strikingly, PN GSCs underwent a MES transition upon serum exposure similar to what is seen with TNFα [20]. The MES GSCs also showed a further shift towards a higher MES metagene (Figure 3A). Gene set enrichment analyses of genes induced by serum differentiation showed similarities to clinical MES GBM signatures as well as normal human MSCs (Figure 3B) [2, 3]. Conversely, the signatures of the serum exposed GSCs showed inverse correlations with TCGA PN, classical (CL) and neural (NL) signatures.

Recently, we have shown that NF-κB signaling activates master transcription factors that mediate MES transition in response to microenvironmental cues [20, 35]. To examine if serum induced MES differentiation utilizes these transcriptional nodes, we performed western blotting analyses. As shown in Figure 3C, phosphorylation of serine 536 on p65 form of NF-κB was induced upon serum culture as well as downstream master TFs STAT3, and TAZ, but not C/EBP-β (Figure 3C). Culturing in serum also dramatically induced CD44 expression in PN GSCs (11 and 23, Figure 3D), whereas MES GSCs retained high CD44 expression even under serum differentiating conditions (data not shown). Thus, exposure to serum readily induces a MES transition as evidenced by gene expression signatures, the expression of the master transcription factors and MES cell surface marker CD44 in PN GSCs demonstrating the plastic nature of these cell types.

**Figure 4:**
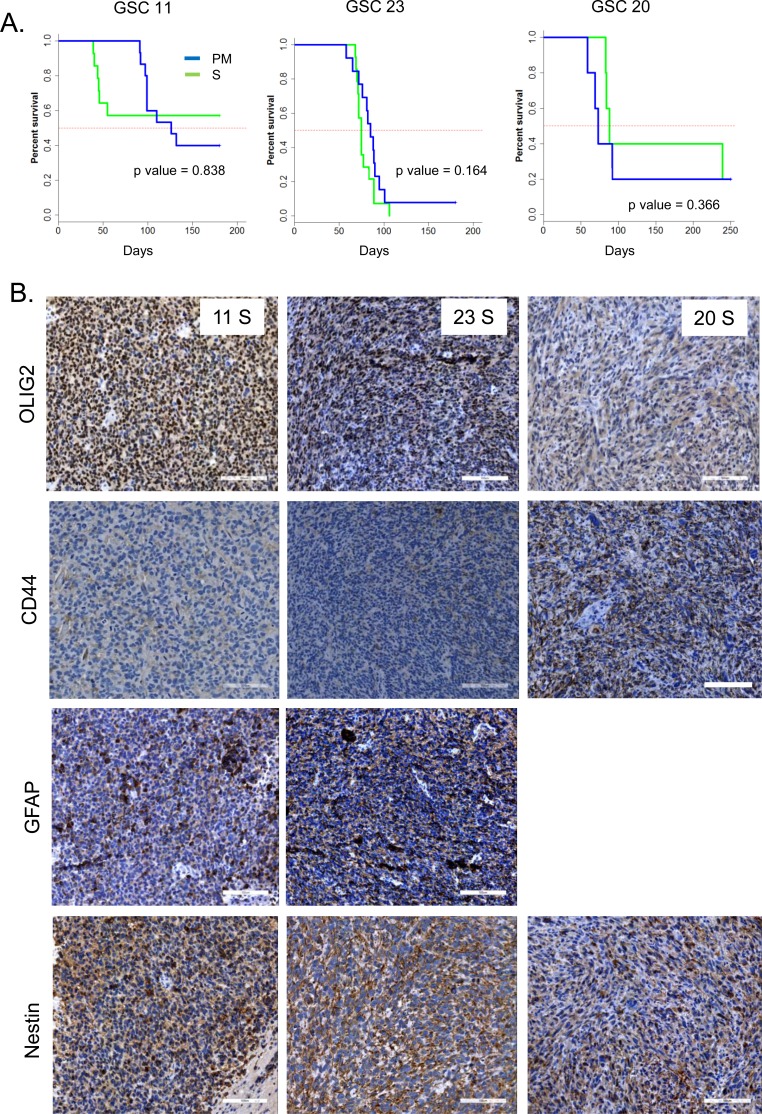
**A.** Kaplan Meier curves show the survival of mice implanted with GSCs after culturing in PM, or S. *p* values were determined by log rank test. **B.** Representative IHC images of GSC xenografts are shown. Scale bar = 100 μM.

Finally, implanting these cells directly after long term serum culture intracranially into mice demonstrated no significant differences in survival in GSCs 23 and 20, but shortened survival in mice implanted with serum cultured GSC11 without significance (Figure 4A). Despite strong MES differentiation induced in culture, tumors generated from serum cultured GSCs failed to maintain the MES subtype *in vivo* as evidenced by high OLIG2 and lack of CD44 expression in these tumors (Figure 4B). These results indicate that under long-term serum culture, GSCs undergo reversible MES differentiation, but retain tumor initiation potential.

## DISCUSSION

In this study we demonstrate that 1) the self-renewal and tumorigenic properties of GSCs are retained despite serum-induced expression of neural differentiation markers and 2) PN GSCs upon serum exposure display a MES pattern of gene expression.

A similar study by Lee et al, using acutely dissociated cells from human tumors grown in serum showed lack of tumorigenic potential while matched cultures grown as neurospheres were highly tumorigenic [10]. Similarly, bone morphogenetic proteins (BMPs)— BMP2, BMP4, and BMP7, and all-trans RA have been shown to inhibit tumor initiation by promoting astorglial and neuronal differentiation in GBM cells [26-29]. Reprogramming strategies using overexpression of the transcription factor neurogenin also impede self-renewal and tumorigenesis in GSCs by promoting neuronal differentiation [36, 37]. Some of these approaches differ significantly from our study in that we have used established GSCs (in contrast to acute tumor dissociation) that may account for differences in the observed phenotype. While one potential concern regarding these differences is that long-term culturing induces artifactual changes in the GSC biology, several previous studies have examined this question and shown that long term culture of GSCs does not alter their genetic alterations compared to their parental tumors [10]. Despite these differences, differentiation as a therapeutic strategy is achievable if we can reprogram these cells fully into astrocytic or neuronal lineages perhaps by blockade of MES differentiation using inhibitors of STAT3 and/or NF-κB signaling.

Alternatively, GSCs use oncogenic pathways to maintain stem cell programs which in turn may cause resistance to complete differentiation. For example, resistance to differentiation of cancer stem cells isolated from a mouse model of GBM that lack p53 and PTEN has been shown to contribute to tumorigenicity due to persistent c-myc activity. It remains to be tested if c-myc levels are induced in human GSCs as well, since this oncogene has also been shown to be a serum response gene [34]. A recent study using a large cohort of human GBM samples showed moderate to high levels of expression of human neural stem cell marker nestin and committed progenitor markers like Olig2, GFAP, Tuj1 and Dcx (neurons), but only few cells expressed terminally differentiated markers such as MBP (for oligodendrocytes) and NeuN (for neurons) [38]. Furthermore a defective Myt1L-A2BP1 axis, that is required to promote terminal neuronal differentiation, was seen in GBM. Collectively, these studies demonstrate that oncogenic pathways present in GSCs may contribute to resistance to terminal differentiation.

This study is a significant extension of our recent report that stemness and gene expression signatures of GSCs are plastic entities that can be influenced by immune cells and other tumor microenvironmental factors [3, 39, 40]. At this time, we cannot pinpoint the factor/s that contribute to MES differentiation in serum, given that serum is complex mixture of growth factors, cytokines, and hormones many of which can activate NF-kB and other master transcription factors of MES differentiation. However, recent studies have shown that serum derived lysophosphatidic acid, and sphin-gosine 1-phosphophate act through G protein coupled receptors to activate YAP/TAZ [41]. We will undertake similar approaches in the future to identify serum-derived factor/s that promote MES differentiation in GSCs to identify novel therapeutic targets.

In summary, the present study shows that established GSCs undergo aberrant differentiation, expressing markers of neural lineages while simultaneously retaining tumor initiating capacity in response to culturing in serum. Furthermore, a shift to a MES signature is associated with this aberrant differentiation. At a minimum, these findings suggest that differentiation toward glio-neuronal lineages could be associated with a parallel MES transition that can interfere with complete terminal differentiation and that inhibition of the drivers of MES differentiation could rescue this process.

## MATERIALS AND METHODS

### Isolation of GSCs

Tumor tissue was obtained from patients who were undergoing surgical resection and were diagnosed with a WHO grade IV astrocytoma (GBM or gliosarcoma). All tissues were obtained from patients who granted written consent according to an IRB approved protocol. Within two hours after surgery the tissues were mechanically and enzymatically (0.25% trypsin/0.04%EDTA; cat no: 25200056; Invitrogen) dissociated, and passed through a 70μm cell strainer (cat no: 352350; BD Falcon) to produce a single cell suspension. Cells were cultured in neurosphere media consisting of DMEM/F12 (cat no:10-090-CV; Cellgro) supplemented with EGF 20ng/ml (cat no: GF144; Chemicon), bFGF 20ng/ml (cat no: GF003AF-MG; Chemicon), 2% B-27 (cat no: 17504-044; Invitrogen), 100U/ml penicillin/streptomycin (cat no: 10378-016; Invitrogen) and 2mM l-glutamine (cat no: 25030-081; Gibco BRL) in uncoated plastic petri-dishes (cat no: 430167; Corning). After two to four weeks, free floating neurospheres were collected by centrifugation and dissociated with Accutase (cat no: A6964; Sigma) and mechanical disruption. Neurospheres were thereafter routinely cultured in the above mentioned neurosphere media, with dissociation to single cells every two to three weeks. Cultures that did not re-form neuropheres after three passages were considered unsuccessful, while cultures that continued to self-renew and form neurospheres after three passages were considered successful.

### Differentiation of GSCs

For short-term differentiation, neurospheres were collected by centrifugation, and dissociated into single cells with accutase. The number of viable cells was determined by trypan-blue exclusion method and plated at a density of 20,000-100,000 cells onto poly-d-lysine coated 12 mm coverslips (cat no: 354086; BD biosciences) or coated petri dishes (cat no: 354468, 354469; BD biosciences) for 7-14 days. Differentiation media consisted of either a) neurosphere media without mitogens, EGF and bFGF (termed basic media, BM), b) basic media with 2μM all *trans*-retinoic acid (RA) or c) DMEM/F12 with 2% or 10% FBS (cat no: 16000-044; Invitrogen). Media was changed every 48 hours. After 7-14 days in differentiation conditions, cells were fixed with 4% paraformaldehyde for immunocytochemistry or lysed with 0.5% NP-40 lysis buffer for western blot analyses [35]. For long term differentiation, cells were cultured and passaged as adherent cells in 10% serum for a period up to 90 days.

### Tumorigenicity evaluation by orthotopic transplantation and immunohistochemistry

Orthotopic transplantation of GSCs were carried out by using implantable guide-screw system as described previously [42]. Briefly, four to six weeks old nude mice (strain nu/nu; Harlan Sprague-Dawley Inc.), each weighing 20-30g were used. Single cell suspensions from different culture conditions (proliferative neurosphere conditions, 10% serum differentiated, or RA differentiated) were injected intracranially at either 2×10^4^ or 2×10^5^ cells per mice. At least four animals were used in each group. Animals were sacrificed at the time of development of neurological symptoms or cachexia. Whole brains from sacrificed mice were embedded in paraffin blocks. Hematoxylin and eosin staining and immunohistochemistry were performed on 10μM-thick paraffin embedded cryostat sections as previously described [35]. Staining was visualized using the DAKO Envision kit according to the instructions of the manufacturer (DAKO, CA). The following primary antibodies were used: Mouse α-human nestin (cat no: MAB5326; Chemicon), mouse α-β III Tubulin (cat no: MAB1637; Millipore), rabbit α-cow GFAP (cat no: Z0334; Dako), rabbit α-Olig2 (cat no: 18953; IBL Co, Japan), mouse α-CD44 (cat no. 3570, Cell Signaling), rabbit α-Sox2 (cat no: ab12052; Abcam), mouse α-actin (cat no: CP01; Calbiochem), and mouse α-vimentin (cat no: CP01; Calbiochem). Secondary antibodies used for immunofluorescence were: fluorescein conjugated goat α-mouse IgG and Texas Red-conjugated goat α-rabbit IgG (DAKO). For western blot experiments, HRP conjugated goat α-rabbit, and goat α-mouse (cat no: 170-6515/6516; Bio-Rad) were used.

### Microarray and bio-informatics analysis

Reference datasets for human MSCs and NSCs were downloaded [43-45]. Microarray data was processed as previously described [20]. To determine the PN and MES metagene scores, first an average expression value for MES or PN genes for each GSC or reference was generated, using a union of the respective Phillips and Verhaak MES and PN genesets. The two average MES and PN values were then z-score corrected among the plotted samples.

## SUPPLEMENTARY MATERIAL FIGURE


