# Supplementary material for: Aberrant mesenchymal differentiation of glioma stem-like cells: implications for therapeutic targeting
Source: Oncotarget. 2015 Aug 19;6(31):31007–17. doi: 10.18632/oncotarget.5219 (PMC4741584; doi:10.18632/oncotarget.5219)
Supplement: Supplementary file 1 [file oncotarget-06-31007-s001.pdf]

Aberrant mesenchymal differentiation of glioma stem-like cells: implications for therapeutic targeting

Supplementary Material

A.

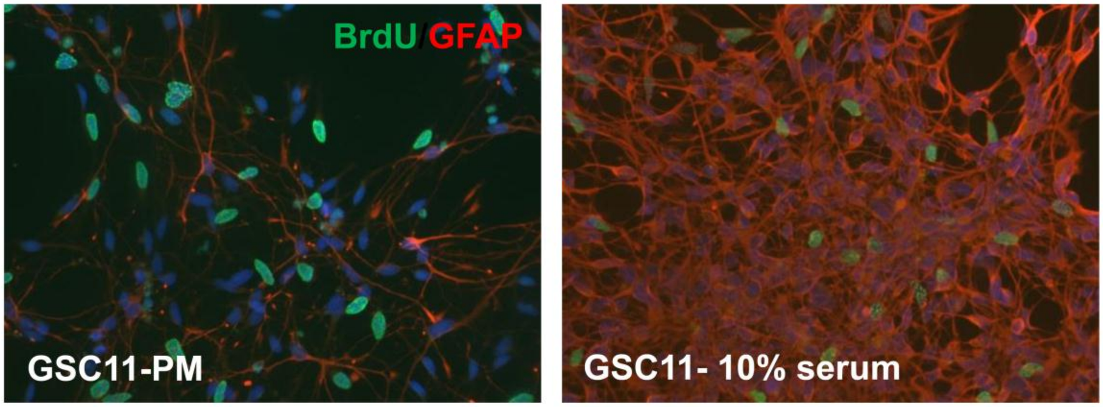

B.

GSC	# of cells	Treatment	# of animals	Median survival (days)	Logrank vs. Control (p-value)
GSC 23	200,000	Control	4	70	
		Retinoic acid	4	67	0.31
		10% serum	4	64	1.00
	20,000	Control	4	78.5	
		Retinoic acid	4	83.5	0.38
		10% serum	4	76.5	0.67
GSC 11	200,000	Control	4	48.5	
		Retinoic acid	4	43.5	0.01
		10% serum	4	49	0.42
	20,000	Control	4	66.5	
		Retinoic acid	4	63	0.81
		10% serum	4	72.5	0.26

C.

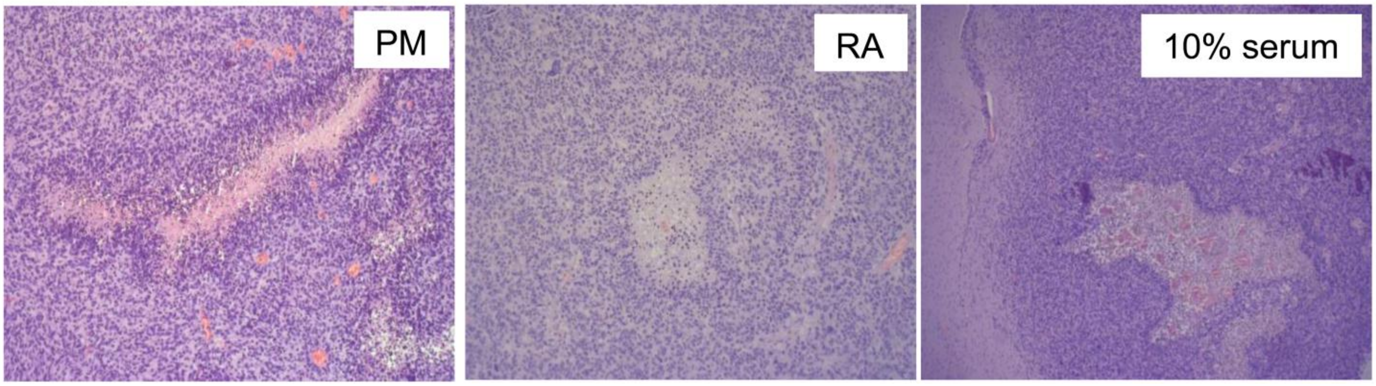

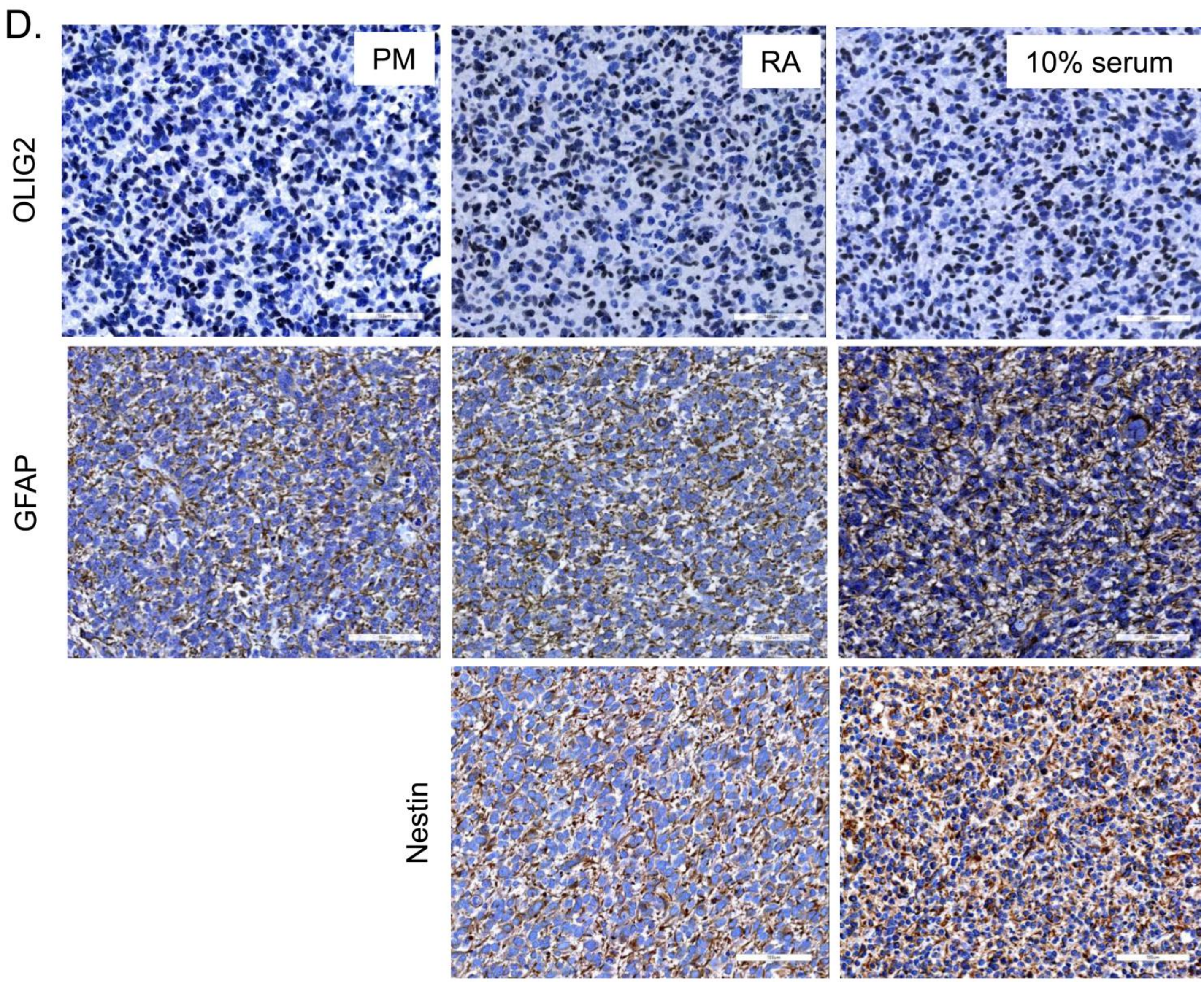

Supplementary Figure 1: A. Representative immunofluorescent images of BrdU (green) and GFAP (red) in GSC11cultured under various conditions. **B.** Survival analysis of animals implanted with 2 x 10^4 and 20 x 10^4 cells after exposure to retinoic acid or 10% serum. *p* values were determined by log rank test. **C.** Hematoxylin and eosin staining of xenograft tumors derived from GSCs subject to various differentiation conditions. **D.** Representative IHC images of GSC xenografts are shown. Scale bar = 100 μM.
